# Mild shading promotes sesquiterpenoid synthesis and accumulation in *Atractylodes lancea* by regulating photosynthesis and phytohormones

**DOI:** 10.1038/s41598-022-25494-7

**Published:** 2022-12-15

**Authors:** Xiuzhi Guo, Qiang Li, Binbin Yan, Yuefeng Wang, Sheng Wang, Feng Xiong, Chengcai Zhang, Yan Zhang, Lanping Guo

**Affiliations:** State Key Laboratory and Breeding Base of Dao-Di Herbs, Resource Center of Chinese Materia Medica, China Academy of Chinese Medical Sciences, Beijing, 100700 China

**Keywords:** Ecology, Secondary metabolism

## Abstract

*Atractylodes lancea* rhizome (AR) has high medicinal and economic value. A previous study has reported that the accumulation of sesquiterpenoids in AR has obvious advantages under bamboo canopy. A concrete shade value to promote the cultivation of high-quality AR has not been established. In this study, 80% shading was screened at six different light intensities (100%, 80%, 60%, 40%, 20%, 7%), and the mechanism was explored in terms of photosynthetic efficiency and phytohormones levels. The results indicated that the total sesquiterpenoid content of 80% mild shading increased by 58%, 52%, and 35%, respectively, compared to 100% strong light in seedling, expansion, and harvest stages and increased by 144%, 178%, and 94%, respectively, compared with 7% low light. The sesquiterpenoids hinesol and β-eudesmol contributed approximately 70% to the differential contribution ratio between mild shading and strong light (100%) or between mild shading and low light (7%). Furthermore, HMGR, DXR, and FPPS genes, which regulate sesquiterpenoid synthesis, were significantly upregulated in 80% mild shading. Transpiration rate; the intercellular CO_2_ concentration; net photosynthetic rate; and levels of jasmonic acid, abscisic acid, and gibberellin were strongly correlated (r > 0.85) with sesquiterpenoid accumulation. Cis-acting elements responding to light and phytohormones were present within the promoter regions of HMGR, DXR, and FPPS. Therefore, 80% shading promotes the synthesis and accumulation of sesquiterpenoids in AR by regulating photosynthetic efficiency and phytohormone production, thereby promoting transcriptional expression.

## Introduction

Atractylodis Rhizoma in Chinese pharmacopoeia is derived from the dry rhizome of *Atractylodes lancea* (Thunb.) DC. Atractylodis Rhizoma have several functions, namely, drying dampness and strengthening the spleen, eliminating pollution and turbidity, eliminating wind dampness, and clarifying the eyes in Traditional Chinese Medicine (TCM) applications^1^. Atractylodis Rhizoma is also one of the main materials in the prevention of COVID-19 infection^2,3^. The major volatile oils in *Atractylodes lancea* rhizome (AR) are the sesquiterpenoids hinesol, β-eudesmol, and atractylone, and the polyacetylene atractylodin, which are acknowledged as the four major medicinal components, as well as the marker compounds, of the quality evaluation of AR^4^. Thus, the higher the volatile oil content, the better the clinical efficacy of AR^5^.

Light is an essential factor that affects the growth, development, and metabolism of plants during their life cycles. Many studies have demonstrated that the accumulation of secondary metabolites can be affected by changes in light intensity. For example, the content of camptothecin in the leaves of *Camptotheca acuminata* seedlings increases as the degree of shading increases; however, it decreases in excessive shading^6,7^. Flavonoids accumulate in *Anoectochilus roxburghii* to a greater extent when the light intensity is low^8^. The synthesis of isorhamnetin can be induced when the light intensity is increased from low light above a certain threshold^9^. *A. lancea* varieties can be found in environments under a wide range of light conditions, according to a survey of wild resources^10^. However, previous studies have reported that bamboo-shaded or maize-shaded *A. lancea* contains more medicinal compounds than that grown in environments without the shade of other plants^11,12^. Shading is a key ecological factor that affects the accumulation of volatile oils in AR. Gu et al. showed that a shading value of 72.5% promotes the accumulation of volatile oils, but the physiological and biochemical indicators of plants are yet to be defined^13^. Sun et al. and Wang et al. detected the antioxidant enzymes and the photosynthetic parameters of *A. lancea* under different shading conditions, but the experimental period was very short and the results of volatile oils were not reported^14,15^. Presently, wild *A. lancea* is a rare resource, while cultivated *A. lancea* has become a major source of AR in production settings^16^. To promote the stable and sustainable production of high-quality AR in the future, establish a concrete shading value to guide the cultivation of high-quality *A. lancea*, and the corresponding mechanism of shading, which enhances the accumulation of volatile oils, also requires further investigation simultaneously.

Photosynthesis ensures life activities, and it is one of the most important metabolic processes. Photosynthetic efficiency is affected by various factors such as light, drought, and temperature, which in turn affect primary and secondary metabolic efficiency^14,17,18^. In many studies, photosynthetic efficiency is reflected by the intercellular CO_*2*_ concentration (C_i_), transpiration rate (T_r_), net photosynthetic rate (P_n_), and stomatal conductance (G_s_)^19,20^. More efficient photosynthesis can generate better plant qualities than those with poor physiological traits^21^. Phytohormones are the key endogenous signals of plant cells in response to changes in the external environment, and they are not only inducers of sesquiterpenoid biosynthesis^22,23^, but also the products of sesquiterpenoid biosynthesis such as abscisic acid (ABA). The expression of key enzymes in artemisinin biosynthesis can be regulated by jasmonic acid (JA), and this process requires light^24^. Therefore, to understand the mechanism of shading that regulates sesquiterpenoid accumulation, the effects of photosynthesis and the changes in plant hormone levels to light intensity are worthy of attention.

In this study, by testing six different light intensity conditions, including 100%, 80%, 60%, 40%, 20%, 7% of natural sunlight, we examined the effects of light intensity on the quality and the yield of AR, The reasons for the formation of high-quality AR were analyzed in terms of photosynthetic efficiency and phytohormone levels. The experiment was carried out in the field for one year, and the goals of this study were as follows: (1) to determine the shade value for the cultivation of high-quality *A. lancea*; (2) to understand the effects of different light intensities on photosynthetic efficiency and phytohormone levels in cultivated *A. lancea*; and (3) to determine the relationship between volatile oils, photosynthetic parameters, and phytohormones under different light intensities.

## Results

### Mild shading facilitates sesquiterpenoid accumulation and growth in *Atractylodes lancea* rhizome

**Figure 1:**
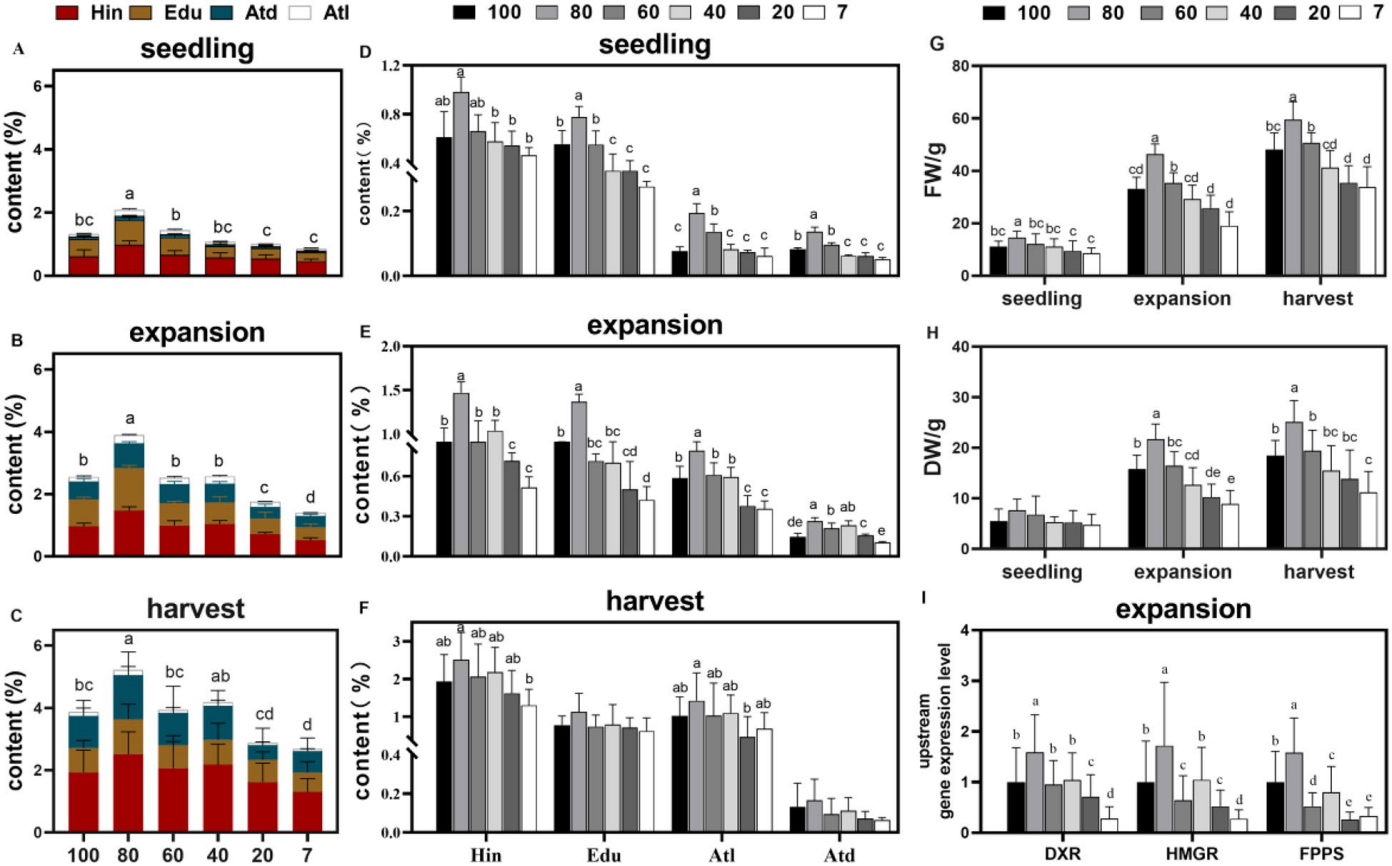
Effects of different light intensities on the accumulation of total volatile compounds and each component (Hin, Edu, Atl, Atd) in the *A. lancea* rhizome in seedling (**A**,**D**), expansion (**B**,**E**), and harvest (**C**,**F**) stages. Effects of different light intensities on the biomass, namely, the fresh weight (FW) (**G**) and the dry weight (DW) (**H**) and the expression of upstream genes encoding key enzymes involved in sesquiterpenoid biosynthesis during the expansion stage (**I**). Lower-case letters represent significant differences (one-way ANOVA, *p* < 0.05).

To determine a concrete shading value for the production of high-quality and high-yielding AR, we examined the major compounds, including the sesquiterpenoids hinesol (Hin), β-eudesmol (Edu), and atractylone (Atl), and the polyacetylene atractylodin (Atd), as well as the biomass of AR at different growth stages (Fig. 1A–C) under various light intensities. The sum of these four volatile oils as the total volatile oil content was subsequently analyzed. The results revealed that the accumulation of volatile oils was significantly different (p < 0.05) across seedling, expansion, and harvesting stages under different light intensities. The common features at the three life stages were that the total volatile oil content was the highest under 80% mild shading and the lowest under 7% low light intensity. At 80% mild shading, the total volatile oil content in seedling, expansion, and harvest stages increased by 58%, 52%, and 35%, respectively, compared to 100% strong light. Thus, excessive light inhibited the accumulation of volatile oils. Compared to 7% low light, the increased accumulation of volatile oils at 80% mild shading reached 144%, 178%, and 94% from seedling to harvesting stages. We found that the weaker the light intensity (20% and 7%), the lower the volatile oil accumulation. In addition, the biomass indicators of fresh and dry weights (Fig. 1G,H) indicated that the response to different light intensities yielded trends that were consistent with volatile oil accumulation. Thus, both quality and yield of AR can be achieved under 80% mild shading.

To further investigate the component that contributes the most to the total volatile oil accumulation due to changes in light intensity, individual compounds were analyzed at different life stages (Fig. 1D–F). The results revealed that each compound was significantly affected by changes in light intensity at different stages (Fig. 1D–F). Statistically, the sum of the difference contribution ratio (DCR) of the three sesquiterpenoids Hin, Edu, and Atl was greater than 90% of the total variation at any stage of growth, with less than 10% of Atd. Of these, Hin and Edu contributed approximately 70% to the total variation (Table S-1). Therefore, the effect of different light intensities on the accumulation of Hin and Edu was higher than that of Atl and Atd. Sesquiterpenoids, especially Hin and Edu, could reliably determine the variations in the total volatile oil content. Compared to 100% strong light, Hin increased by 60%, 52%, and 30% and Edu increased by 40%, 60%, and 45% at 80% mild shading, respectively, from seedling to harvesting stages. Compared to 7% low light, Hin increased by 111%, 185%, and 92%, and Edu increased by 182%, 224%, and 81% at 80% mild shading, respectively, from seedling to harvesting stages. In addition, we also measured the levels of the key enzyme genes of sesquiterpenoid biosynthesis during the expansion stage of AR. The expression levels of HMGR, DXR, and FPPS in the mevalonic acid (MVA) pathway and methylerythritol phosphate (MEP) pathway were measured by qRT-PCR. The results revealed that the expression of these genes under different light intensities significantly differed among the groups (Fig. 1I). The expression of each gene was the highest under 80% mild shading, that is, greater than 100% strong light, and the lowest under 7% low light. The differential expression patterns of these genes under different light intensities is consistent with sesquiterpenoid accumulation. Thus, the expression analysis further confirmed that light intensity affects sesquiterpenoid accumulation.

### 80% mild shading enhances photosynthetic efficiency in *A. lancea*, whose trend was affected by changes in light intensity, consistent with sesquiterpenoid accumulation

**Figure 2:**
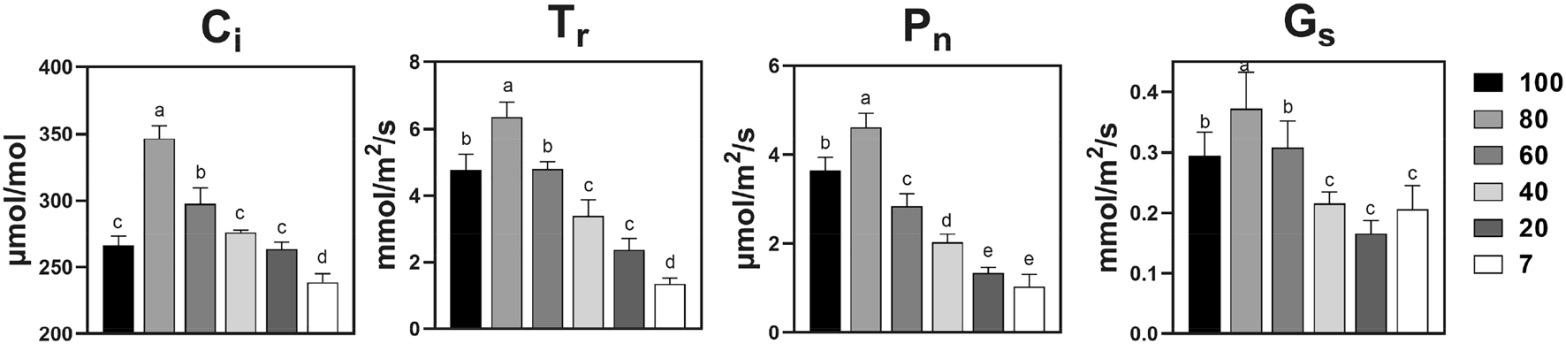
Effects of different light intensities on photosynthetic parameters, namely, the intercellular CO_2_ concentration (C_i_), transpiration rate (T_r_), net photosynthetic rate (P_n_), and stomatal conductance (G_s_) in *A. lancea*. Lower-case letters represent significant differences (one-way ANOVA, *p* < 0.05).

The intercellular CO_2_ concentration (C_i_), transpiration rate (T_r_), net photosynthetic rate (P_n_), and stomatal conductance (G_S_) as gas exchange parameters were significantly different at the different light intensities. The C_i_, T_r_, P_n_, and G_s_ values of 80% mild shading were the highest (Fig. 2), increasing by 30%, 33%, 27%, and 27%, respectively, compared to 100% strong light and increasing by 45%, 369%, 347%, and 81%, respectively, compared to 7% low light. The results showed that between 100% strong light and 80% mild shading, the differences of the four photosynthetic parameters are close to 30%. While between 7% low light and 80% mild shading, the changes of T_r_, P_n_ are far higher than C_i,_ G_s_. As the degree of shade increased, light utilization decreased starting at 80% light intensity. The results showed that mild shading promotes photosynthetic efficiency in *A. lancea*, and the trend of variation is consistent with sesquiterpenoid accumulation, which is affected by changes in light intensity.

### 80% mild shading significantly enhances the phytohormones ABA and GA_3_ in ***A. lancea***, whose trend was affected by changes in light intensity, consistent with sesquiterpenoid accumulation

**Figure 3:**
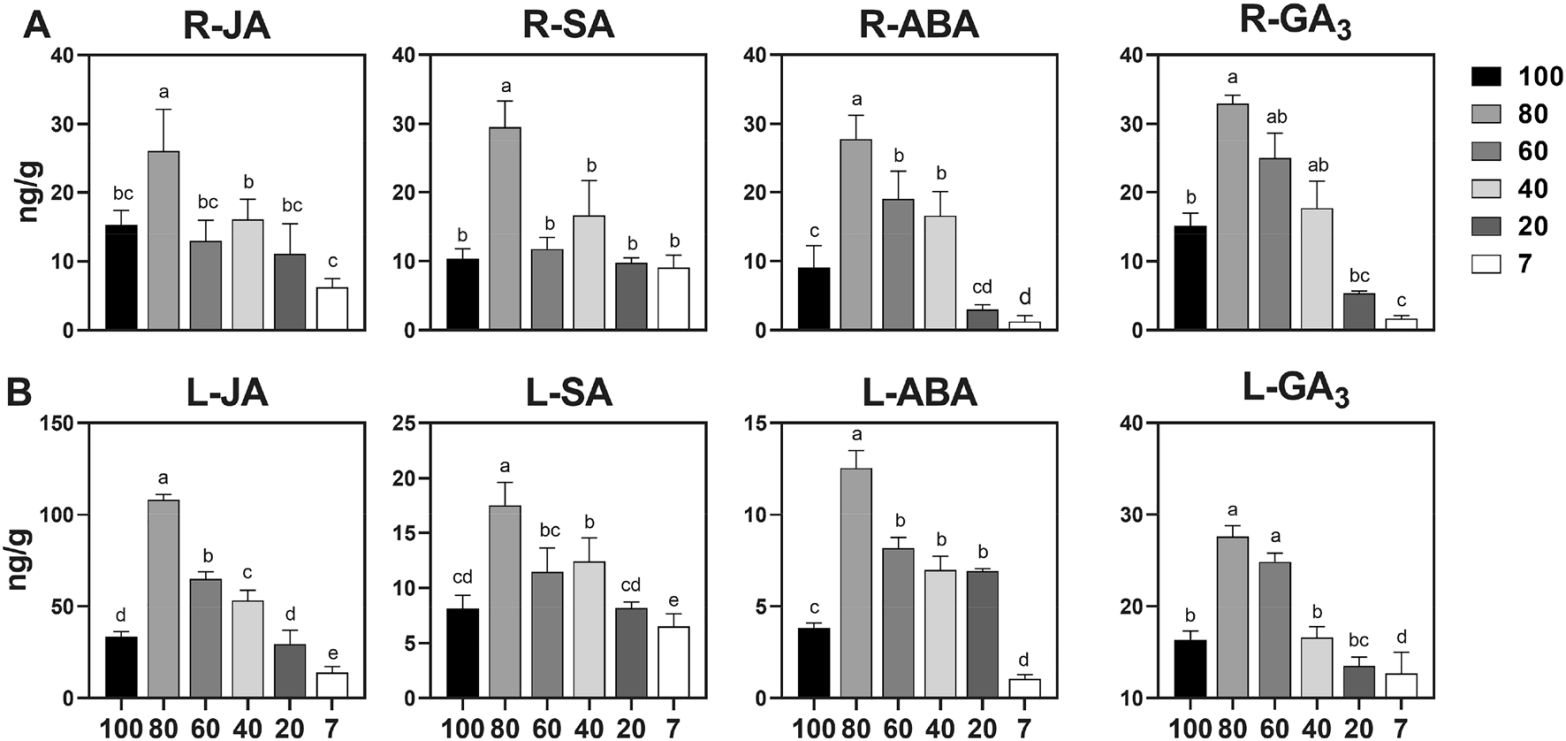
Effects of different light intensities on the phytohormones jasmonic acid (JA), salicylic acid (SA), abscisic acid (ABA), gibberellin (GA_3_) in *A. lancea* roots (**A**) and leaves (**B**). Lower-case letters represent significant differences (one-way ANOVA, *p* < 0.05).

Phytohormones play important roles in signal transduction and secondary metabolism in plants. We measured the levels of common phytohormones in root (R) and leaf (L), as shown in Fig. 3. Data analysis found that the levels of JA, salicylic acid (SA), ABA, and gibberellin (GA_3_) under 80% mild shading were significantly (*p* > 0.05) higher than that of 100% and 7% light intensities, regardless of the plant part (Fig. 3). Furthermore, JA, SA, ABA, and GA_3_ levels in 80% mild shading were 1.7, 2.9, 3.1, and 2.2-fold higher than those in 100% strong light, and 4.2, 3.2, 22.9, 19.8-fold higher than those in 7% low light in root (Fig. 3A). And JA, SA, ABA, and GA_3_ levels in 80% mild shading were 4.2, 3.2, 21.2, and 19.8-fold higher than those in 100% strong light, and 7.8, 2.9, 11.8, 2.2-fold higher than those in 7% low light in leaves (Fig. 3B). The results showed that between 100% strong light and 80% mild shading, the phytohormone change folds of ABA and GA_3_ are higher than other phytohormones in leaves. While between 7% low light and mild shading, in root the phytohormone change folds of ABA and GA_3_ are higher than other phytohormones. Therefore, under strong light stress, ABA and GA_3_ in leaves were more responsive, while under weak light stress, ABA and GA_3_ in roots were more responsive. Other phytohormones content also fluctuated with different light intensity changes, but the fluctuation range was relatively small. In addition, all phytohormone levels gradually decreased with weakening light intensity starting at 80% light intensity (Fig. 3). The results indicated that the changes in the phytohormone levels are consistent with those of sesquiterpenoid accumulation.

In addition, we compared the phytohormone response levels in root and leaf. When the phytohormone change fold (HCF) of roots to leaves was equal to 1, the hormone response level was similar in both root and leaf. When the HCF was higher than 1, the hormone response level was higher in root than in leaf, indicating that hormonal stress in roots is more sensitive to changes in light intensity. By contrast, when the HCF was lower than 1, the hormone response level was lower in root than in leaf, indicating that hormonal stress in leaves is more sensitive to changes in light intensity. Table 1 shows that JA was more sensitive to light intensity changes in leaves than in roots, while ABA was higher responseive to light intensity changes in roots than in leaves.

**Table 1:** Hormonal changes folds (HCF) in roots compared to leaves under the influence of different light intensities.

Phytohormone	100%	80%	60%	40%	20%	7%
JA	0.46	0.24	0.20	0.30	0.38	0.45
SA	1.27	1.68	1.02	1.34	1.20	1.39
ABA	2.38	2.21	2.33	2.38	0.43	1.19
GA3	0.92	1.19	1.01	1.07	0.40	0.13

### Ci, Tr, and Pn in photosynthesis; GA_3_ and ABA in root; and JA in leaf were strongly correlated with the accumulation of each sesquiterpenoid

**Figure 4:**
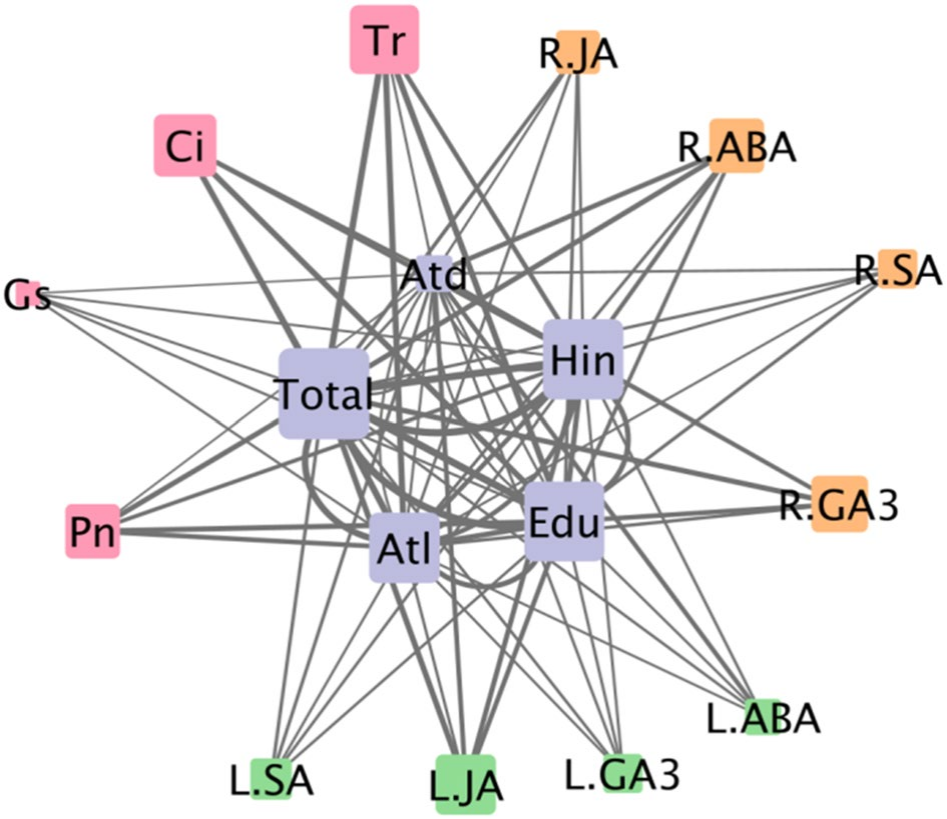
Pearson correlation network diagram between volatile oils and physiological and biochemical indexes. Different colors of nodes represent volatile oils (purple), photosynthetic parameters (pink), phytohormones in leaves (green), and phytohormones in roots (orange). The size of each node indicates the amount of the correlated index. The width of each edge represents the correlation. As the width of each edge increases, the correlation coefficient is greater (r > 0.85 and *p*< 0.05). The connectivity degree is indicated by the size of the symbols.

The relationships among various physiological and biochemical factors and volatile oils were assessed by Pearson correlation coefficients under different light intensities. As shown in Table S-2, most indexes were significantly correlated with the volatile oil content (*p* < 0.01). The correlation coefficient represents the strength of the correlation between the two indexes. Correlation coefficients greater than 0.85 were used as the screening criteria. As shown in Fig. 4, the values of C_i_, T_r_ and P_n_ were strongly correlated with the sesquiterpenoids hinesol and β-eudesmol (r > 0.85). From the correlation network, we found that R-ABA, R-GA_3_ and L-JA showed the highest correlation with the accumulation of sesquiterpenoids. Based on the results of photosynthetic efficiency test, phytohormone test and Pearson correlation analysis, Tr and Pn in photosynthesis, GA3 and ABA in root, and JA in leaf that are not only significantly responsive to light intensity changes but also significantly related to sesquiterpenes are screened out.

### Promoter regions of HMGR, DXRs, and FPPS contain both light and phytohormone cis-acting regulatory elements

HMGR, DXR, and FPPS have been reported as key enzyme genes involved in biosynthesis of sesquiterpenoids. Therefore, we analyzed the cis-acting elements within the upstream promoter regions (2000 bp) of the three genes which had been selected for qRT-PCR analysis. The relevant sequences are shown in Fig. S-1. The results indicated that HMGR, DXR, and FPPS promoter regions included several light and phytohormone regulatory elements (Table 2), confirming that sesquiterpenoid genes may co-regulated by light and phytohormones.

**Table 2:** The cis-acting elements related light and phytohormone were contained in the promoter upstream 2000 bp region of HMGR,DXR,FPPS.

	Light	JA	SA	ABA	GA
HMGR	ATCT-motif	CGTCA-motif	–	ABRE	–
	Box 4	TGACG-motif	–	–	–
	GT1-motif	–	–	–	–
	MRE	–	–	–	–
	TCT-motif	–	–	–	–
DXR	Box 4	CGTCA-motif	TCA-element	ABRE	GARE-motif
	G-box	–	–	–	–
	LAMP-element	–	–	–	–
	MRE	–	–	–	–
FPPS	AE-box	CGTCA-motif	TCA-element	–	–
	ATCT-motif	–	–	–	–
	B-box4	–	–	–	–
	GA-motif	–	–	–	–
	GT1-motif	–	–	–	–
	TCT-motif	–	–	–	–
	chs-CMA1a	–	–	–	–
References	^25–28^	^29–31^	^32^	^33,34^	^35^

## Discussion

### Mild shading facilitates the accumulation of volatile oils and growth in *A. lancea*

Synthesize multiple results showed that Tr and Pn may be the key extrinsic factors affecting the synthesis and the accumulation of the sesquiterpenoids hinesol and β-eudesmol. We know that photosynthesis is an important indicator of growth and development as well as stress resistance in plants^36,37^. This study showed that Pn, which reflects plant organic matter accumulation, was the highest under 80% mild shading. In addition, AR also accumulates more biomass under this condition. The substances produced by photosynthesis far exceeded the substances consumed by the plant for its own life activities, indicating that it grew well. The 100% strong light provided more light energy than 80% mild shading, but the biomass of 100% strong light was lower than that under 80% mild shading. This can be explained by the fact that high light and high temperature in the summer cause severe leaf burns, and photosynthetic structures are irreversibly damaged^14^, leading to an imbalance of Tr and a decline of Pn. According to Farquhar and Sharkey, who commented on the reduced photosynthetic rate, when Pn and Gs decrease, if Ci also decreases, this indicates that the reduction of Gs is the main reason for the reduced photosynthetic rate^38^. Meanwhile, under the long-term regulation of shaded environments, stomatal density can decrease, leading to the decline of Gs^39^. Furthermore, Gs is the main factor of Tr intensity and Pn efficiency. Combined with the results of this study, under conditions of < 80% mild shading, with the increase of shading degree, photosynthetic efficiency was mainly affected by stomatal limitations. Studies have demonstrated that low light can reduce photosynthesis by inhibiting Ci and Gs to suppress secondary metabolic processes such as phycocyanin synthesis and yield in crops such as wheat^40,41^. Therefore, under 80% mild shading, *A. lancea* maintained the best photosynthesis state, and it was more capable of resisting the fluctuations of the external environment to produce more sesquiterpene secondary metabolites in a long-time environment. In addition to having better resistance to the environment at 80% mild shading, more organic substances accumulated in AR, thereby providing the elements needed for energy production such as acetyl coenzyme A, malonyl coenzyme A, and ATP^42^ using for sesquiterpenoid biosynthesis.

### Light-hormone interactions regulate key enzyme gene expression in sesquiterpenoid synthesis and further influence sesquiterpenoid accumulation

As physiological and biochemical indicators, phytohormones reflect the intrinsic expression of plant life activities. The Pearson correlation results showed that the accumulation of sesquiterpenoids is strongly related to the changes of multiple hormones at different light intensities. Our findings are consistent with the results that the promoters of three key enzyme genes in the sesquiterpenoid synthesis pathway contain multiple cis-acting elements of phytohormones. Chuanchao and colleagues demonstrated that JA can up-regulate the expression of sesquiterpenoid genes through fungal induction; it also has functional interactions with SA in plant defense^43^. On the other hand, ABA and GA_3_ up-regulate DXS and HMGR genes under bacterial induction and promote the accumulation of volatile compounds^44,45^. It was also found that blocking the production of just one hormone did not completely inhibit sesquiterpenoid production^43^, suggesting that sesquiterpenoid synthesis is not dependent on a single signaling event and that multiple phytohormones work together to regulate their synthesis. These findings reveal that multiple phytohormones showed good correlation with the accumulation of sesquiterpenoids. A previous molecular study demonstrated a relationship between light and several hormone signaling pathways via signaling integrators (PIFs, HY5)^46^. It has also been demonstrated that the function of JA in artemisinin is dependent on the presence of light^47^. Therefore, light may be the basic condition required to induce changes in phytohormone levels. However, *A. lancea* maintains different phytohormone levels, and their responses are the same at different light intensities. The levels were higher in mild shading, and lower in low light shading. This may be related to the growth inhibitory mechanism used by *A. lancea* under the stress of strong light or weak light for a long time to maintain a longer lifespan. In summary, the results of this study suggest that mild shade induces changes in the phytohormones ABA, and GA_3_, JA, which regulate the expression levels of key enzyme genes for sesquiterpenoid synthesis and ultimately influence the changes in bioactive components.

Our findings revealed that ABA decreases under strong light, which is inconsistent with the results of a previous study that reported ABA unaffected by light signals^48^. Under long-term high light conditions, the leaves of *A. lancea* showed burning and yellowing as well as poor growth. Abscisic acid is a hormone that triggers leaf abscission, so ABA can be used as an indicator of the abscission of injured leaves, indicating that ABA levels change with plant growth and development. In addition, it was found that the content of ABA in roots was significantly higher than that in leaves. Currently, ABA is synthesized at several sites, such as roots and leaves, and translocated to its sites of action, which includes guard cells^49^. Given that plants have multiple ABA transporters, ABA transport is dynamically regulated under various growth conditions, which regulates root system expansion^50,51^.

In addition, we found that the promoters of key enzyme genes that regulate the synthesis of sesquiterpenoids contain numerous light response elements. Several studies have demonstrated that light can directly regulate the synthesis of isoprene, the precursor of sesquiterpenoids^52^. Therefore, in the future, studies should focus on the significance of light, which directly regulates the synthesis of sesquiterpenoids via the downstream factors of the light response.

## Conclusion

In conclusion, 80% mild shading is a proper shade condition suitable for guiding stable and sustainable production of high-quality AR. It promotes the accumulation of total volatile oils mainly by regulating the biosynthesis of the sesquiterpenoids hinesol and β-eudesmol. Mild shading promotes the synthesis and accumulation of the medicinal compound in *A. lancea* via integration and regulation of photosynthesis and phytohormones. In addition, light and phytohormones are important internal and external factors affecting plant growth, development, and metabolism, although the direction of molecular research of *A. lancea* has been slow. Thus, this study provides a solid foundation upon which future studies can be designed. In the future, the relationship between photosynthesis (Tr, Ci, Pn), multiple hormones (ABA, GA_3_, JA), and sesquiterpenoids (hinesol, β-eudesmol) at the molecular level should be studied, laying the foundation for the regulation and the improvement of AR quality.

## Materials and methods

All plant experiments were performed in accordance with relevant guidelines and regulations.

### Field experiment, sampling, and sample preparation

The field experiment was conducted from November 2016 to November 2017 at the GAP production base of Jiufang Pharmaceutical Company, Yuexi County, Anhui Province, China, and *A. lancea* seedlings were developed from the buds of the second-year rhizome by vegetative propagation. The germplasm identification of the seedlings was confirmed by Professor Lanping Guo from the National Resource Center for Chinese Materia Medica, China Academy of Chinese Medical Sciences. Healthy and uniformly sized *A. lancea* seedlings were planted in an experimental field located on a flat plot at an altitude of 870 m with a row spacing of 0.5 m and a plant spacing of 0.3 m. Different levels of shade above the seedlings were created in the greenhouse using materials of different densities such as white gauze and sunshade net. There were six experimental groups with five replicates in each group. A TR-72U light quantum instrument (Thermo Fisher Scientific, Waltham, MA, USA) was used to determine the light intensity of the different shading treatments every 10 min from 8:00 to 17:00 on sunny days, and the full daylight intensity without any shade was measured, which served as a control. The percentage of light intensity in the other groups relative to the full light intensity was used as an indicator of the light intensity in the growth environment. The experimental groups were as follows: 100% (full light group), 80% light intensity group (two layers of white gauze), 60% light intensity group (four layers of white gauze), 40% light intensity group (one layer of sunshade net), 20% (one layer of white gauze and one of sunshade net, respectively), and 7% (two layers of sunshade net). An area up to 30 cm above the ground surrounding the greenhouse was enclosed to ensure the smooth flow of air.

### Extraction and determination of volatile compounds at different developmental stages

**Figure 5:**
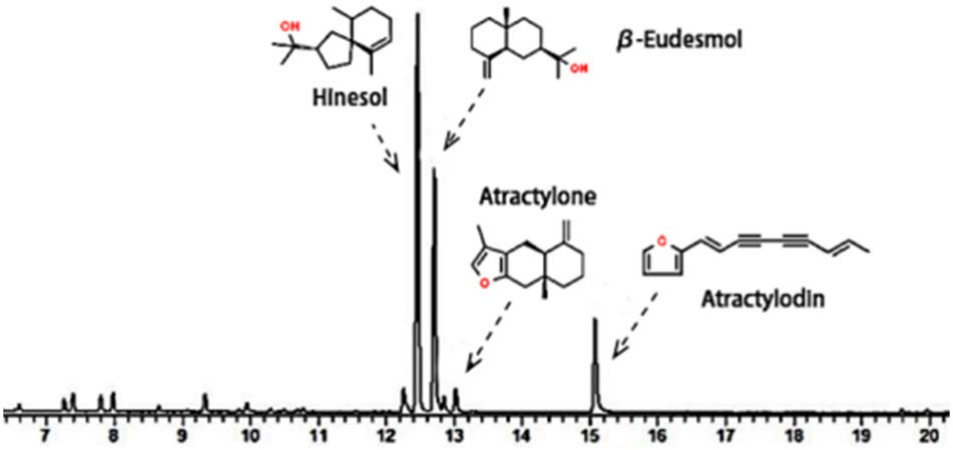
Typical GC–MS chromatogram of the classical volatile oils present in *A. lancea*.

The concentrations of volatile oils in the rhizome of *A. lancea* were determined at different developmental stages, including seedling, expansion (period of rapid growth of rhizome), and harvest. First, the fresh rhizome samples were dried in an oven at 40 °C to a constant weight and weighed. The dried samples were crushed into a powder with a ball mill, passed through a 60-mesh sieve, and used to determine the chemical composition of *A. lancea*. Approximately 500 mg of each powdered sample was obtained and placed in a 50-mL centrifuge tube. After adding 25 mL of n-hexane (purity ≥ 95%, Beijing Chemical Works, Beijing, China), ultrasonic extraction was conducted at 40 kHz for 30 min. The samples were centrifuged at 3000 r min^− 1^ for 10 min, and the supernatants were collected. The pellet residues were suspended, and 25 mL of n-hexane was continuously added and extracted. This process was repeated, and after two rounds of centrifugation, the supernatants were combined, mixed well, and finally diluted with n-hexane to 50 mL. After filtration through an organic membrane, the samples were analyzed by gas chromatography–mass spectrometry (GC–MS)^53^. A TRACE 1310 GC instrument coupled to a TSQ 8000 mass spectrometer (Thermo Fisher Scientific) was used. An Agilent DB-5MS capillary column (0.25 mm × 30 m, 0.25 μm) was used; the carrier gas was helium gas, and the flow rate was 1 mL min^− 1^. The injection mode was split flow (ratio 50:1), the injection port temperature was 240 ℃, and the injection volume was 1 μL. The temperature was increased to 120 °C for the first 2 min, and then to 240 °C at a rate of 5 °C min^-1^, and then maintained at 240 °C for 5 min. The other parameters were as follows: EI (ionization voltage: 70 V, ion source 230 ℃, quadrupole temperature: 150 ℃) and MSD data acquisition mode (scanning ion range m/z 40–500). Figure 5 shows the GC–MS chromatograms of the four major volatile compounds in *A. lancea* ^53^.

### Expression of genes encoding key enzymes involved in the biosynthesis of sesquiterpenoids in the expansion stage

The fresh rhizome samples of the expansion period, a mixture of roots, and leaves of *A. lancea* were stored in a small liquid nitrogen tank in the field and used for RNA extraction. Approximately 50 mg of leaves and roots were mixed and RNA was extracted with the TransZol UP Plus RNA Kit (TransGen Biotech Co., Beijing, China). The RNA was reverse-transcribed to obtain cDNA using the TransScript All-in-One First-Strand cDNA Synthesis SuperMix for qPCR (TransGen Biotech Co.). Primers of genes encoding key enzymes involved in the sesquiterpenoid biosynthesis pathway (DXR, HMGR, and FPPS) were designed based on our transcriptome database (Table S-3). Real-time quantitative PCR (qRT-PCR) was conducted according to the instructions of the TransStart Top Green qPCR SuperMix Kit (TransGen Biotech Co.), and EF-1α served as the internal reference gene.

### Measurement of the biomass of *A. lancea* at different developmental stages

Rhizome biomass, including the fresh weight (FW) and dry weight (DW) of *A. lancea* at seedling, expansion, and harvest stages was measured. The aerial parts and roots of the harvested *A. lancea* samples were removed, and the weight of the underground rhizomes was measured. There were ten replicates for each treatment group.

### Determination of the photosynthetic parameters of *A. lancea* leaves at the expansion stage

The leaf photosynthetic parameters of *A. lancea*, including the intercellular CO_*2*_ concentration (C_i_), transpiration rate (T_r_), net photosynthetic rate (P_n_), and stomatal conductance (G_s_), were determined under different light intensities using a Li-6400 portable photosynthesis instrument on a sunny day from 9:00 to 11:00 a.m. and 2:00 to 4:00 p.m. when plants were in the expansion stage. *A. lancea* leaves fully exposed to light and at the same positions were randomly selected. For each group, five plants were selected, and measurements were taken from two leaves on each plant. Each leaf was measured three times.

### Determination of the endogenous hormone content in roots and leaves of *A. lancea* at the expansion stage

The contents of JA, ABA, SA, and GA_3_ were determined in the roots and leaves of *A. lancea*^53^, and each group had three replicates. After removing the surface dirt, the roots and leaves were immediately ground in a liquid nitrogen dry ice bath, weighed, and added to a 2-mL centrifuge tube. Next, 0.5 mL of isopropyl alcohol:water:HCl (2:1:2) extract was added to the centrifuge tube and mixed (4 ℃, 100 r min^−1^) for 30 min, and 1 mL of dichloromethane was then added to the centrifuge tube and mixed for 30 min. The samples were centrifuged (4 ℃, 13,000 r min^−1^) for 5 min, and 0.9 mL of the organic phase layer (middle layer) was absorbed and blow-dried with nitrogen. Next, 200 µL of a 50% methanol–water solution was added, followed by centrifugation (4 °C, 13,000 r min^-1^) for 15 min. The supernatant was then extracted for UPLC-MS/MS analysis. Chromatography was conducted with a Waters ACQUITY UPLC BEH C18 column (2.1 mm × 100 mm, 1.7 µm). The flow phase was 0.05% formic acid water–acetonitrile (Thermo Fisher Scientific). The linear gradient elution was as follows: 0–0.3 min, 10% acetonitrile; 0.3–3 min, 10–60% acetonitrile; 3–6 min, 60–95% acetonitrile; and 6–6.2 min, 10% acetonitrile. The flow rate was 0.6 mL min^-1^, and the column temperature was 4 ℃. The sample tray temperature was 4 ℃, the injection volume was 10 µL, and the analysis time was 7 min. The mass spectrometry conditions were as follows: the ionization mode was electrospray ion source (ESI), the scanning mode was negative ion scanning, the monitoring method was multi-response monitoring (MRM), the pressure of the air curtain was 30 psi, the ionization voltage was − 4500 V, the temperature of the ion source was 550 ℃, the spray gas pressure was 50 psi, and the auxiliary heating gas pressure was 50 psi. Data processing was conducted with Multi Quant Software.

### Analysis of cis-acting elements in the predicted promoter regions of HMGR, DXR, FPPS genes that regulate sesquiterpenoid biosynthesis

Using the genomic data from this experimental platform, the upstream predicted promoter regions of HMGR, DXR, and FPPS was obtained by TBtools, and the potential cis-acting elements were predicted using Plantcare Software (http://bioinformatics.psb.ugent.be/webtools/plantcare/html/).

## Data analysis

Microsoft Excel 2016 and SPSS v26.0 Software (IBM, Armonk, NY, USA) were used for statistical and correlation analyses. The results were expressed as means ± standard deviations (SD). One-way analysis of variance (ANOVA), followed by the Fisher’s protected least significance difference test, was performed to determine the main effects. The figures in the manuscript were created with GraphPad Prism Software and Adobe Illustrator CS6. The graphs for Pearson correlation network diagram were prepared with Cytoscape Software.

## Supplementary Information

Supplementary Information.

## Data Availability

The original contributions presented in this study are included in the article/supplementary material, and further inquiries can be directed to the corresponding authors.

## References

[1] Zhang WJ (2021). Atractylodis Rhizoma: A review of its traditional uses, phytochemistry, pharmacology, toxicology and quality control. J. Ethnopharmacol..

[2] Yang Y, Islam MS, Wang J, Li Y, Chen X (2020). Traditional Chinese medicine in the treatment of patients infected with 2019-new coronavirus (SARS-CoV-2): A review and perspective. Int. J. Biol. Sci..

[3] Zhao Z (2021). Prevention and treatment of COVID-19 using traditional Chinese medicine: A review. Phytomedicine.

[4] Yu DQ (2019). Microscopic characteristic and chemical composition analysis of three medicinal plants and surface frosts. Molecules.

[5] Deng A (2016). Advances in studies on chemical compositions of*Atractylodes lancea* and their biological activities. China J. Chin. Materia Med..

[6] Yang W, Jun DS, Feng YX (2004). Effects of light intensity on secondary metabolite camptothecin production in leaves of *Camptotheca acuminata* seedlings. J. Acta Ecol. Sin..

[7] Ma X (2015). Growth, physiological, and biochemical responses of *Camptotheca acuminata* seedlings to different light environments. Front. Plant Sci..

[8] Niu H (2020). Effects of light intrnsity on the growth, physiological properties and medicinal components of*Anoectochilus roxburghii*. J. Plant Resour. Environ..

[9] Zheng L, Ma Z, Xiao Y (2016). Effects of different light intensities on growth and secondary metabolite synthesis of *Anoectochilus formosanus*. Anhui Agric. Bull..

[10] Sun Y (2008). Biomass sructure analysis of *Atractyloddes lancea* in different ecological environments. China J. Chin. Materia Med..

[11] Zhang Y (2015). Effects of different niches on the growth and four volatile oils of *Atractylodes lancea*. Chin. J. Tradit. Chin. Med..

[12] Peng Z (2021). Diverse intercropping patterns enhance the productivity and volatile oil yield of *Atractylodes lancea* (Thunb.) DC.. Front. Plant Sci..

[13] Gu Y, Feng X, Xia B (2008). Effect of light intensity on rhizome biomass and volatile oil content of *Atractylodes Lancea*. Jiangsu Agric. Sci. J..

[14] Wang Y (2020). Effect of strong light stress on growth, physiological and biochemical and gene expression of key enzymes in *Atractylodes lancea*. Chin. J. Exp. Tradit. Med. Formulae.

[15] Sun, Y., Wang, Y., Gu, W. & Chao, J. Effects of strong light stress on photosynthetic fluorescence parameters of *Atractylodes lancea*. *North. Hortic.* (22), 112–116 (2021).

[16] Gao X, Bai R, Wei J, Zhao C (2022). Research progress on germplasm resources and cultivation technology of *Atractylodes lancea*. J. Chengde Med. Univ..

[17] Zhang A (2021). Effect of drought on photosynthesis, total antioxidant capacity, bioactive component accumulation, and the transcriptome of *Atractylodes lancea*. BMC Plant Biol..

[18] Li M, Chao J, Gu W, Hou H (2015). Effects of high-temperature stress on photosynthetic characteristics and physiological indexes of *Atractylodes lancea* (Thunb.) DC. from different producing areas. J. South. Agric..

[19] Liang YF, Yi JN, Wang KC, Xue Q, Sui L (2019). Response of growth and photosynthetic characteristics of Polygonatum cyrtonema to shading conditions. Zhongguo Zhong Yao Za Zhi.

[20] Shan Z (2018). Physiological and proteomic analysis on long-term drought resistance of cassava (*Manihot esculenta* Crantz). Sci. Rep..

[21] He J, Chua EL, Qin L (2020). Drought does not induce crassulacean acid metabolism (CAM) but regulates photosynthesis and enhances nutritional quality of Mesembryanthemum crystallinum. PLoS ONE.

[22] Liu C (2012). Influence of four plant hormones on the accumulation of sesquiterpenoids in leaves of *Artemisia annua* L.. J. Ningxia Med. Univ..

[23] Liao, Y. *Molecular Mechanism of JA Signaling Pathway Involved in the Regulation of Agarwood Sesquiterpene Biosynthesis* (Peking Union Medical College, 2015).

[24] Hao X (2017). Transcriptome analysis of genes associated with the artemisinin biosynthesis by jasmonic acid treatment under the light in *Artemisia annua*. Front. Plant Sci..

[25] Roy S, Choudhury SR, Singh SK, Das KP (2012). Functional analysis of light-regulated promoter region of AtPolλ gene. Planta.

[26] Liang J (2021). Identification of HvLRX, a new dehydration and light responsive gene in Tibetan hulless barley (*Hordeum vulgare* var. nudum). Genes Genomics.

[27] Wang Z (2022). Rapeseed (*Brassica napus*) Mitogen-activated protein Kinase 1 enhances shading tolerance by regulating the photosynthesis capability of Photosystem II. Front. Plant Sci..

[28] Yang, P.* Screening and Analysis of LRE Binding Proteins for BrCHS Promoter* (Northeast Forestry University, 2012).

[29] Mason HS, DeWald DB, Mullet JE (1993). Identification of a methyl jasmonate-responsive domain in the soybean vspB promoter. Plant Cell.

[30] Rouster J, Leah R, Mundy J, Cameron-Mills V (1997). Identification of a methyl jasmonate-responsive region in the promoter of a lipoxygenase 1 gene expressed in barley grain. Plant J..

[31] Xu Z (2018). Glycinebetaine biosynthesis in response to osmotic stress depends on jasmonate signaling in watermelon suspension cells. Front. Plant Sci..

[32] Mou S (2013). Functional analysis and expressional characterization of rice ankyrin repeat-containing protein, OsPIANK1, in basal defense against *Magnaporthe oryzae* attack. PLoS ONE.

[33] Uno Y (2000). Arabidopsis basic leucine zipper transcription factors involved in an abscisic acid-dependent signal transduction pathway under drought and high-salinity conditions. Proc. Natl. Acad. Sci. U S A.

[34] Kochan E (2019). Abscisic acid regulates the 3-hydroxy-3-methylglutaryl CoA reductase gene promoter and Ginsenoside production in *Panax quinquefolium* hairy root cultures. Int. J. Mol. Sci..

[35] Li W (2019). SmGRAS1 and SmGRAS2 regulate the biosynthesis of Tanshinones and phenolic acids in *Salvia miltiorrhiza*. Front. Plant Sci..

[36] Wu W (2022). Effect of shading on photosynthetic characteristics and nutrient accumulation of *Aglaonema commutatun* seedling. Subtrop. Agric. Res..

[37] Zheng, J.* et al*. Functional characterization of SbER10_X1 on regulating photosynthesis and biomass of forage sorghum. *Acta Prataculturae Sin.* 1–11 (2022). <https://kns.cnki.net/kcms/detail/62.1105.s.20220929.1226.037.html>

[38] Farquhar GD, Sharkey TD (1982). Stomatal conductance and photosynthesis. Ann. Rev. Plant Physiol..

[39] Li SL (2022). Responses of leaf stomatal and mesophyll conductance to abiotic stress factors. J. Integr. Agric..

[40] Yang H (2020). Photosynthetic base of reduced grain yield by shading stress during the early reproductive stage of two wheat cultivars. Sci. Rep..

[41] Zhu H (2017). Effects of low light on photosynthetic properties, antioxidant enzyme activity, and anthocyanin accumulation in purple pak-choi (*Brassica campestris* ssp. Chinensis Makino). PLoS ONE.

[42] Zhang WJ (2020). Pharmacodynamic material basis of traditional Chinese medicine based on biomacromolecules: A review. Plant Methods.

[43] Ren CG, Dai CC (2012). Jasmonic acid is involved in the signaling pathway for fungal endophyte-induced volatile oil accumulation of *Atractylodes lancea* plantlets. BMC Plant Biol..

[44] Wang XM (2015). Involvement of abscisic acid and salicylic acid in signal cascade regulating bacterial endophyte-induced volatile oil biosynthesis in plantlets of *Atractylodes lancea*. Physiol. Plant.

[45] Zhou JY, Li X, Zhao D, Deng-Wang MY, Dai CC (2016). Reactive oxygen species and hormone signaling cascades in endophytic bacterium induced essential oil accumulation in *Atractylodes lancea*. Planta.

[46] Lau OS, Deng XW (2010). Plant hormone signaling lightens up: integrators of light and hormones. Curr. Opin. Plant Biol..

[47] Hao X (2019). Light-induced artemisinin biosynthesis is regulated by the bZIP transcription factor AaHY5 in *Artemisia annua*.

[48] Galvez-Valdivieso G (2009). The high light response in Arabidopsis involves ABA signaling between vascular and bundle sheath cells. Plant Cell.

[49] Kuromori T, Seo M, Shinozaki K (2018). ABA transport and plant water stress responses. Trends Plant Sci..

[50] Chen K (2020). Abscisic acid dynamics, signaling, and functions in plants. J. Integr. Plant Biol..

[51] Yoshida T, Christmann A, Yamaguchi-Shinozaki K, Grill E, Fernie AR (2019). Revisiting the basal role of ABA—Roles outside of stress. Trends Plant Sci..

[52] Rodríguez-Concepción M (2004). Distinct light-mediated pathways regulate the biosynthesis and exchange of isoprenoid precursors during Arabidopsis seedling development. Plant Cell.

[53] Li Q (2018). Effect of different light quality on the growth, antioxidant enzyme activity and volatile oil content of *Atractylodes lancea*. Chin. J. Exp. Tradit. Med. Formulae.

