# Supplementary material for: Mild shading promotes sesquiterpenoid synthesis and accumulation in Atractylodes lancea by regulating photosynthesis and phytohormones
Source: Sci Rep. 2022 Dec 15;12:21648. doi: 10.1038/s41598-022-25494-7 (PMC9755305; doi:10.1038/s41598-022-25494-7)
Supplement: Supplementary file 1 — Supplementary Information. [file 41598_2022_25494_MOESM1_ESM.docx]

Supplementary Tables and Figures

| growth stage | component | 80% vs 100% | | 80% vs 7% | |
| --- | --- | --- | --- | --- | --- |
|  |  | DV(%) | DCR(%) | DV(%) | DCR(%) |
| seedling | Hin | 28 | 49 | 61 | 42 |
|  | Edu | 17 | 29 | 59 | 41 |
|  | Atl | 9 | 15 | 16 | 11 |
|  | Atd | 4 | 7 | 10 | 7 |
|  | total | 58 | 100 | 145 | 100 |
| expansion | Hin | 20 | 38 | 68 | 38 |
|  | Edu | 20 | 38 | 68 | 38 |
|  | Atl | 8 | 15 | 31 | 17 |
|  | Atd | 5 | 9 | 11 | 6 |
|  | total | 52 | 100 | 178 | 100 |
| harvest | Hin | 15 | 42 | 45 | 47 |
|  | Edu | 9 | 26 | 19 | 20 |
|  | Atl | 10 | 29 | 27 | 29 |
|  | Atd | 1 | 3 | 4 | 4 |
|  | total | 35 | 100 | 95 | 100 |

**Table S-1**. Analysis of the differential contribution ratio of each component (Hin, Edu, Atl, Atd) and total volatile oil (total) of 80% light intensity compared with 100% strong light and 7% low light, respectively, in seedling, expansion, and harvest stages. The percentage variance of each compound between 80% and 100% or 80% and 7% was regarded as the differential value (DV); The ratio of the DV of each component to the DV of the total volatile components is considered as the differential contribution ratio (DCR). Hin (hinesol); Edu (β-eudesmol); Atl (atractylone); Atd (atractylodin); total (total volatile oil)

|  | Hin | Edu | Atl | Atd | Total |
| --- | --- | --- | --- | --- | --- |
| Ci | 0.881** | 0.917** | 0.799** | 0.835** | 0.919** |
| Tr | 0.879** | 0.925** | 0.930** | 0.701** | 0.937** |
| Pn | 0.854** | 0.898** | 0.883** | 0.541* | 0.896** |
| Gs | 0.666** | 0.736** | 0.700** | 0.480* | 0.719** |
| R.JA | 0.803** | 0.828** | 0.812** | 0.702** | 0.847** |
| R.SA | 0.797** | 0.832** | 0.666** | 0.704** | 0.817** |
| R.ABA | 0.865** | 0.850** | 0.827** | 0.875** | 0.896** |
| R.GA3 | 0.856** | 0.879** | 0.830** | 0.850** | 0.902** |
| L.JA | 0.891** | 0.889** | 0.822** | 0.876** | 0.920** |
| L.SA | 0.815** | 0.849** | 0.748** | 0.859** | 0.859** |
| L.ABA | 0.826** | 0.732** | 0.668** | 0.890** | 0.805** |
| L.GA3 | 0.760** | 0.826** | 0.725** | 0.811** | 0.820** |
| ** p＜0.01; * p＜0.05 | | | | | |
| **Table S-2.** Pearson correlation coefficients of photosynthetic parameters (Ci, Tr, Pn, Gs), phytohormones (JA, SA, ABA, GA_3_) of leaf (L) and root (R) with volatile oils (Hin, Edu, Atl, Atd, total). Hin (hinesol); Edu (β-eudesmol); Atl (atractylone); Atd (atractylodin); total (total volatile oil) | | | | | |

| primer | sequence(5'-3') |
| --- | --- |
| HMGR-F | CATCTACCTCCTCGGCTT |
| HMGR-R | CTGGCATCTCACAACACT |
| DXR-F | AAGATGTTAAAGTAGCCGATGC |
| DXR-R | GAATGTATGATAGAATGAGGGT |
| FPPS-F | TCTTTGTCTATTCACCGC |
| FPPS-R | TCCAATCTTGCCTATCAC |
| EF-1α-F | CAGGCTGATTGTGCTGTTCTTA |
| EF-1α-R | TGGTGGCA TCCATCTTGT |
| **Table S-3.** Primers of HMGR, DXS, FPPS and EF-1α genes used for real-time quantitative polymerase chain reaction. EF-1α was internal reference gene in *A.lancea* | |

HMGR

gaaagaaaaaatatgagtaaattacagttttggtccctgaggtatatatggtcttgcagtcatcgtccctaaagttcaataattacagttaaggtcactgacgtatccatttcctttcacctttcgtccctgccgttaaaaagtccagttaactttaacagatagagtggtattttcgtctttttatacctt

TGACG-motif

aaggaccaaaactgtaatttattcttttgatttttagtttttaagttaagttttgattttttatttaaattaatttagttttttatttaaattaaaaaca

Box 4

aaaaaacaaaaaacccacccctcccccaccctcgccggccatcccctccggccaacccatcttctccggctacccccaccccttccctaccctcgccgctcatcttctccggctaacccgcacccctcccgtaaacctaatccaaaacctaaatcaaaaccaaaaaatcagttcaattcaaaccgt

ACAT-element

MRE

ARE

aaacttagtccaaaaccttaaatcaattcaattcaagccaccaacccatcgtcaccaccttaaatcaattcaaaccaaaccgtaaacctaatc

ACAT-element

CGTCA-motif

ARE

CAT-box

caaaacctaaaccgtaaacctaatcaattcaattcaagccaccaacccatatctcatttttaaacctcaagtaaacataaaaatgatgctata

MRE

MRE

aagtgaagataaatgttagaaatgaaaacccagcaaaaatcgactcaaatcgagctcgaaatcgacttccatggcgacaagatgacagtg

gttaatcccatagttacgttgatgctaaggactcaaatgaaatcaacgaataaaccttggaatcgaagatatatgtgctaggaactgataaca

GT1-motif

atcaaacgaagtcgaagtgtgaacaaatccccaccgatctcaaaccaacgaattgtgaaaccaacgaagtgtgaaatcaacgaatcccca

ARE

ARE

ccgatctcaccggccattcatttccccaccctcaccacccccctgcaacctcgcaccaccaccaccaccacccccttccccaccctcaccaccccctgcaacctcgcactaccaccacaaaaccctaaatcatttgaaaacaaaataaaacccaaatcaaaccacaaaaccctcacacacggca

ARE

aacctcacccacagaaatcgtatctcaagcaacaaaccatcaaacctaaatcaaaccaaatcaaaaaccctaaattttacactaaataagg

ARE

ARE

MRE

Agaagaagaagaagaagaagaagaagaagaagagggaagaggagggggcgacatcgaaggtccctgtggtggtggtttcttcgccact

CAT-box

gtacctgttgaaatcgaaggtctcggccgtgtaaatcattgggtgcctgtggcccgagagagagagagagagatagagagagatggagaaaatggtggtgccgtcttcagagaaaatggtggtggtggtgtggtggtgtcaacgccggtgccgtgtccagagaacgagagaaggggtcgggcgagggtttccatcatcgacgacgggggaggtcttttagtagggggttgagggttttttttttgttttatttaattttaaagcaaaaagactaata

tgcccctcatctaactgagttttttaacggattgttagctcagggacgaaaggtgaaatgaaattcatacgtcagtgaccttaactgtaattatt

CGTCA-motif

gaaccttatggacgatgactgaaagaccatatatacctcagggatcaaaactgcaatttactcaaaaaatatattgcattaatggtgatggtc

Box 4

gatagtcatttgagggactgaatttgtttttagaagatatttgagggaccgtcatcgtaattttgatgaatgaagacgcggagttcttctggaac

CGTCA-motif

cgcacgtggtacatgtggcttcatgctttcttactgtccttctataaattgctcccgatgccttcattcctccccccacacctcaccctcataaatc

TCT-motif

ABRE

tcttggccaccgccactctctgttatcttaacgccgatcaaaa

CAT-box

DXS

tgatgttgaaactctcctggatcacttcaaaatgggaaaatagtcgtactgtctgacatgcttaaagaatggacgattacacctaacgattacacaagttaataatatataataattataaaacttattcgttaaagaatttttttttcttgataatttagtttaattataaaacttattcgtttaaatacaatttttcttgataatttacgaaatttagttttactaaaaaaaggaatatattaaaataaatattaaaaatgcttgtttatcgaatgggaaattaaattaaaaataattaagagagagagagagagaaatctgttgaccaagtaattgggaaacgcactcgtcaaaacagtaaattattaaacaac

GARE-motif

CGTCA-motif

Aaattatttaaaaaaaaataaaaaaataaaaaaattgaccatattggtttatttaacagcatcatattgggtgattggaatatatggtgtaatgatcccagtagtgcagtgcaggtgttgtagtatttgtctttatttcattttgtttttcctcattgtagctatttcgcaagcttctggcttgttattttaatatatgatgggggtcgaggcgggatgatggcattgccgaagctcttttgcctcgttccctctttttgccgttcaaaaaaaaataaaaaaatcat

TCA-element

aaacgacgctatattttaatgaaaatgcagtccacgagtagacctctttcgccacgagcattaatgtattatcctccttcgtcatcgttcgtcgtc

Box 4

CGTCA-motif

TGA-motif

tccaataaatttttactccgccctacatatatgaatatattccaattaccccctctagttttggaacttactcctgctcacttatttccaaaaacttgcataaccttcccaaaattgtacttgattctttcaaaatctccatctatcaatgcctccattgtttttaactacatttacctacatgtgatctcaac

aatctttgaacaaactcgagtatcctccaagaagacaacaccttcacgattataaattcacaatttccatgttcaatctatcaagtaaaccaaa

ARE

ctaaaccaaaccaaattagaatctcaaaaggggtctggtgaggtgggatgtacacaactttaaccccacccaaaaggatgaagatgttgttt

ARE

ccgtggaaaccctcagctcatggtgatctggcagaaatctgtgtcatgctctcaagtacacggtcttgtgaaaacttgcaaaagaagcatcattcctgcaaagcttcattaggtcagggccaaggcaagggctcgaacactgcgaacctaaatcaactacagactaatccctaccctcgcccctc

MRE

tcaggtcgaagacgcaccctaggcgtgctcagctaccttaatcttgcgcctccaagaacttctgtcagaggtaatgccctcgggtatatctcgcatcaccatgtccccccttaactgcttttcccacgtcttctttgacttacccttcctcctcaccccttctaccatcacgctttctatcattctcaccggc

G-box

tagtatggtcaaaccgccttaatctacactctcttaagtttatttgtaataggggccactcatatctctgcaccaaacacctgttagaaatcctat

CAT-box

ccaactagggaaatttttatttgtatccagaactatcgtttaactttatcataagaagctaatctttgataaagtttacatatttggaaaagagac

LAMP-element

atgggagctattgcagctaaaagccaacctcaaggggtaacaactgtaattttatccatttatggtatactaaccaccatttatagtatactaa

MBS

Ccaccatcggcctacttcttcccttctatttctctgaaacccaactctttaccaccagcagtgattgccgctctgctgctgtcctctttctttggacccttcctataatacccacatagctattcggatttttttcgtctttctctttctactttctgggaatcaagattttactgaaaaattgaaagaaaaat

FPPS

caaagataaaatcatgtttagtgcagtgacacttatttgacaaattgacctcttacctcatgccttatattagtgcttgcgtggggaaacaaaa

TCT-motif

ttgaaaaaagaaaaacaaataaatacccctttcattttaaacagggacaaagacaaaaataacaactttgtattaatggattccaaaatata

Box 4

gcgtcttttttttcaatttctacaaaatagcatgattttagagaaaaaaaattgattttgccgaaaatgtaaaaaaaaaatgcagaaaacacc

LTR

aaaaaatgtacattttttcggcaaaaaaatgattttttcggcattgaacaattttcggcaaaatcatgctattttgtagaatatgaaaaaaatacgctgtattttggaattttttatcacaaaaacgctatatttgtcattgtcccttttaaatacttcctgataatatataacattagaaacttataagct

AE-BOX

aacttaacggcaattgatttttttttattaaaagttgtatggattaatataatcataagtattaaaatgtttgtatgaattaatatatagtgagttt

Box 4

Box 4

gtttttatgccgaataaaagatctaaatggttaaaattactaattcatccattttaaatgaacaaaaaaaaatcataaatctaatacacaatat

ATCT-motif

GT1-motif

atggtaaggtttttaaaaaatatatatataagagcctaatgggtaaagaaatgtttcatttgcttaaactattaagcctttttgttaactttaaat

aaaaaacaaacccttctatatgaattacctaattaaactatacaacattaagctcattaaagaaaaaaacaaacatgtccttaaaatgataaataacaaaaaatcatttttaaaaataaattttagttaatgattatattttagaaattaaaaataaaataattatgtttggaaatataaattgacaaattatatgaaattaatgtaacaaattttgtttcgattcctattttgtataacacgctttcaaatccggtgtctaagatatgatatctcaaacattt

Box 4

aaatacatatgttacttaatgattaatattctgattttgcagaagtgacatcaatatttttagggacaaagacaaaaataacaactttgcgttaa

Box 4

chs-CMA1a

tggattccaaaatatagcgttttttctttcaattttcataaaatagcatgattttggagaaaaaaattgattttgccgaaaatttggaaaagatg

LTR

cggaaaacacaaaaaatgtacattttttcggcaaaaatgtgattttttcggcactgctacagtgattttcggcactgtagcaaaagtataaatg

ggacaaaacaccgtagcaatgccgaaaatttgcatttttgccgaaaaaatgtactttttttacattttcggcaaaattgaaccattttcggcaa

LTR

LTR

LTR

LTR

LTR

aatcatgttattttgtggaatatgaaaaacaatacgctatattttggaatttttgatcacaaaaacgcaatatttgtcattgtcccatatttttaac

TCA-element

tatttaaatgtgaattaagcaagataaacgttctgataaaattattattattttttgcttaatttacatttaaaaagaataaaaatatcgacatcacggcaacaatgtgatcagttttacaagtcattagataaaatagataattaaatgtttataattttatatagattaaacaccaaaaaatgttttga

GA-motif

aaataacttatataaagaaagaattaagacaaaatatattagtattttcgtataatttacccatataaattttaataaaatgaaggacaaaac

agaccccgcagtgttgtgtcattcagcccacagtacagcagcaggatctgtctacaaatacttcctctcgcacccaatctcaacccaccgtcac

CGTCA-motif

ctctctctctctctctctctctctctctctctctctctctctctctctctcctcaccggacaaacaccggcatctgattccaactattcttctctcactgcaaca

**Figure S-1.** The upstream promoter regions (2000 bp) of the three genes HMGR, DXR, and FPPS as key enzyme genes involved in biosynthesis of sesquiterpenoid. light-responsive elements (pink), phytohormone-responsive elements (red), and other response elements (green) with marked function in Plantcare Software are shown. TATA-box (core promoter element), CAAT-box (common cis-acting element in promoter and enhancer regions), cis-element without marked function in Plantcare Software are not shown in the figure.
